# Self-assembly of hexahistidine-tagged tobacco etch virus capsid protein into microfilaments that induce IgG2-specific response against a soluble porcine reproductive and respiratory syndrome virus chimeric protein

**DOI:** 10.1186/s12985-016-0651-y

**Published:** 2016-11-29

**Authors:** Carlos Alberto Manuel-Cabrera, Alba Adriana Vallejo-Cardona, Eduardo Padilla-Camberos, Rodolfo Hernández-Gutiérrez, Sara Elisa Herrera-Rodríguez, Abel Gutiérrez-Ortega

**Affiliations:** Unidad de Biotecnología Médica y Farmacéutica, Centro de Investigación y Asistencia en Tecnología y Diseño del Estado de Jalisco A.C., Normalistas 800, Colinas de la Normal, 44270 Guadalajara, Jalisco Mexico

**Keywords:** Tobacco etch virus, Capsid protein, Virus-like particles, Hexahistidine tag, Adjuvant, Chimeric protein

## Abstract

**Background:**

Assembly of recombinant capsid proteins into virus-like particles (VLPs) still represents an interesting challenge in virus-based nanotechnologies. The structure of VLPs has gained importance for the development and design of new adjuvants and antigen carriers. The potential of Tobacco etch virus capsid protein (TEV CP) as adjuvant has not been evaluated to date.

**Findings:**

Two constructs for TEV CP expression in *Escherichia coli* were generated: a wild-type version (TEV-CP) and a C-terminal hexahistidine (His)-tagged version (His-TEV-CP). Although both versions were expressed in the soluble fraction of *E. coli* lysates, only His-TEV-CP self-assembled into micrometric flexuous filamentous VLPs. In addition, the His-tag enabled high yields and facilitated purification of TEV VLPs. These TEV VLPs elicited broader IgG2-specific antibody response against a novel porcine reproductive and respiratory syndrome virus (PRRSV) protein when compared to the potent IgG1 response induced by the protein alone.

**Conclusions:**

His-TEV CP was purified by immobilized metal affinity chromatography and assembled into VLPs, some of them reaching 2-μm length. TEV VLPs administered along with PRRSV chimeric protein changed the IgG2/IgG1 ratio against the chimeric protein, suggesting that TEV CP can modulate the immune response against a soluble antigen.

**Electronic supplementary material:**

The online version of this article (doi:10.1186/s12985-016-0651-y) contains supplementary material, which is available to authorized users.

## Findings

The structural proteins of some viruses occasionally mimic the three-dimensional nature of an actual virus while lacking the virus genome packaged inside its capsid [1]. These structures, also called virus-like particles (VLPs), apart from bearing self-assembly properties, feature highly ordered structure and surface repetitiveness, making them good candidates for the development of vaccines and epitope presenting platforms [2]. The upsurge of these applications has driven the cloning, expression and purification of virus structural components in a wide range of host systems (reviewed by Zeltins [3]). However, in most cases the self-assembly of viral capsid proteins (CPs) into VLPs still remains a challenge [4].

We previously attempted to explore the potential of *Tobacco etch virus* (TEV) particles as an adjuvant and our findings suggested that TEV induce both humoral and cellular response without the need of any other stimulus [5]. However, the use of plant viral infectious particles poses additional safety and environmental challenges [6]. By 1990s, research on Johnson grass mosaic virus (JGMV) CP led to propose the use of chimeric potyvirus-like particles for epitope carrying or display taking advantage of particle features, as reviewed by Jagadish and others [7]. For the first time, Jagadish and others [8] successfully expressed in a recombinant system (*E. coli*) the JGMV CP that assembled into virus-like particle structures. Since then, the list of VLP assembly from *E. coli*-expressed potyviral CPs has been extended to Potato virus Y (PVY) [9], Plum pox virus [10], Pepper vein banding virus [11], Papaya ringspot virus [12] and TEV [13]. Interestingly, none of these potyviral CPs were expressed as a fusion to a Histidine tag, perhaps based on the rationale that this tag would compromise CP self-assembly. In the current report we investigated whether TEV CP VLPs can be assembled from Histidine-tagged TEV CP, produced and purified at high level from *E. coli* cultures. We also evaluated the potential use of TEV CP VLPs as an adjuvant for a novel porcine respiratory and reproductive syndrome virus (PRRSV) chimeric protein.

**Fig. 1:**
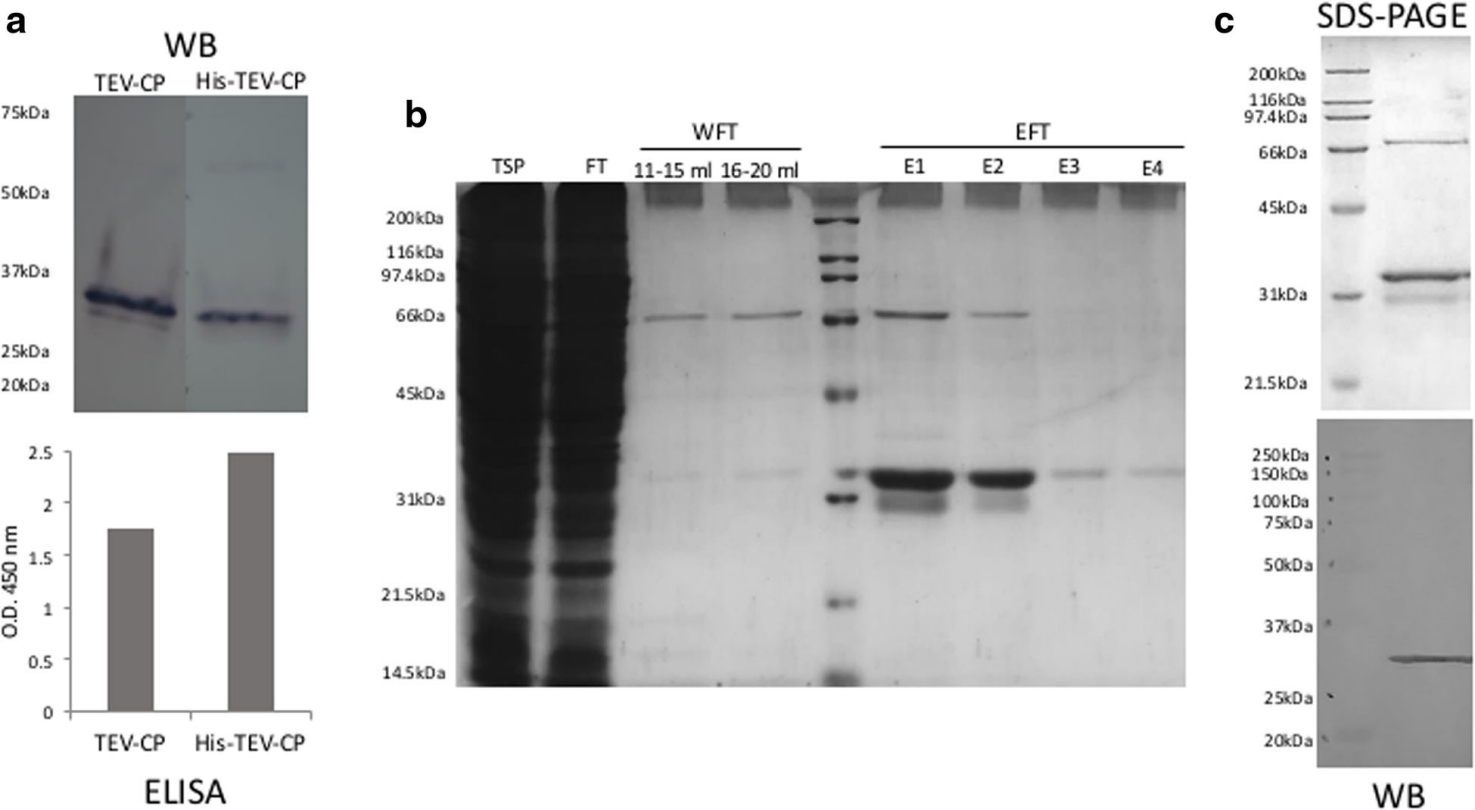
Analysis of TEV CP proteins expression in *E. coli* lysates and purification. The cells were cultured in Terrific Broth medium (Sigma-Aldrich, USA) and grown at 30 °C, until OD_600_ reached 1.0. Overnight induction was performed at 20 °C. 1 mM IPTG was used as inducer and cells were lysed by sonication in 20 mM Tris, pH 8.0 (matrix buffer, MB), 500 mM NaCl. **a** Immune detection analysis of TEV CP proteins using anti-6xHisTag antibody (Roche, Switzerland) for western blot (WB) and anti-TEV antibody (Agdia, USA) for ELISA. **b** Analysis of the purification process of His-TEV-CP by 12% SDS-PAGE loading 10 μl of sample per lane. The purification process was performed using His Trap HP IMAC 1 ml columns (GE-Healthcare, USA). Total soluble protein (TSP) in MB, 500 mM NaCl and 10 mM imidazole was loaded into the column. The flow-through (FT) was collected. Washing steps were performed in MB, 40 mM imidazol, and NaCl gradient (adjusted every 5 ml at 200, 400, 600 and 800 mM). The wash FT (WFT) was collected in 4 fractions, respectively. The proteins were eluted using MB, 100 mM NaCl and 500 mM imidazol. The elution FT (EFT) was collected in 6 fractions of 1 ml each. **c** Analysis of purified His-TEV-CP by 12% SDS-PAGE and WB using anti-His antibody loading 10 μl of sample per lane. The first two fractions of EFT were prepared in MB, 500 mM NaCl, using 3 kDa Amicon Ultra centrifugal filter devices (Merck-Millipore, Germany). Protein concentration was estimated against a BSA curve using Bradford reagent

Two capsid protein versions, TEV-CP and His-TEV-CP, were produced and recovered from soluble fraction of *E. coli* lysates. For generation of the TEV CP expression constructs, *tev cp* gene (GenBank: JX512813.1) modified to contain a glycine codon at 5′- end for facilitating subsequent cloning and codon-optimized for improving *E. coli* expression was synthetically obtained (GenScript, USA). NcoI and XhoI recognition sites were introduced at 5′- and 3′- ends, respectively, by PCR for subsequent ligation into IPTG-inducible expression vector pET28a + (Merck-Millipore, Germany). A stop codon upstream of XhoI recognition site was added in the TEV-CP version, which would result in a protein without the His-tag. The cloning procedures were performed as described previously [14]. The constructs were mobilized to BL21 (DE3) *E. coli* expression strain and IPTG-induced cultures were analyzed for TEV CP expression. Western blot revealed a band corresponding to ~ 31 kDa for each version of the TEV CP (Fig. 1a), similar to the TEV CP estimated mass of 30.2 kDa by CLC Main Workbench (Qiagen, Denmark). As assessed by ELISA, His-TEV-CP expressed at higher level compared to the non-tagged version (Fig. 1a).

For obtaining VLPs from TEV CP soluble non-tagged version, lysates were treated with 4% (w/v) PEG 8000 for 1.5 h at 4 °C with constant shaking and left for 1 h at room temperature (22 ± 2 °C). Then, the sample was centrifuged at 4 °C, for 20 min, at 2,370 × *g* and the precipitate was solubilized overnight in 10 mM Tris-HCl buffer, 300 mM NaCl, 10 mM EDTA, pH 7.4. Samples were centrifuged at 4 °C, for 20 min, at 2,370 × *g* and VLP-enriched supernatants were passed through a 100 kDa MWCO Amicon Ultra-4 filtration centrifugal unit (Merck-Millipore, Germany). The estimated yield of this non-tagged version was 1.2 mg/l of culture, as assessed by densitometric analysis, due to presence of several proteins belonging to the expression host that also precipitated with PEG. On the other hand, the His-tagged version was purified by Ni^2+^ affinity chromatography. Analysis of purification fractions showed recovery of partially purified His-TEV-CP protein (Fig. 1b). Larger and shorter forms of TEV CP were observed in purified preparations of His-TEV-CP version, which might correspond to dimers and proteolysis byproducts, respectively (Fig. 1c). The yield of purified TEV CP was calculated at 10 mg/l of culture, as estimated using Bradford reagent against a BSA curve. Final preparations were stable for months when maintained at 4 °C in matrix buffer (see Fig. 1 legend) with 500 mM NaCl.

**Fig. 2:**
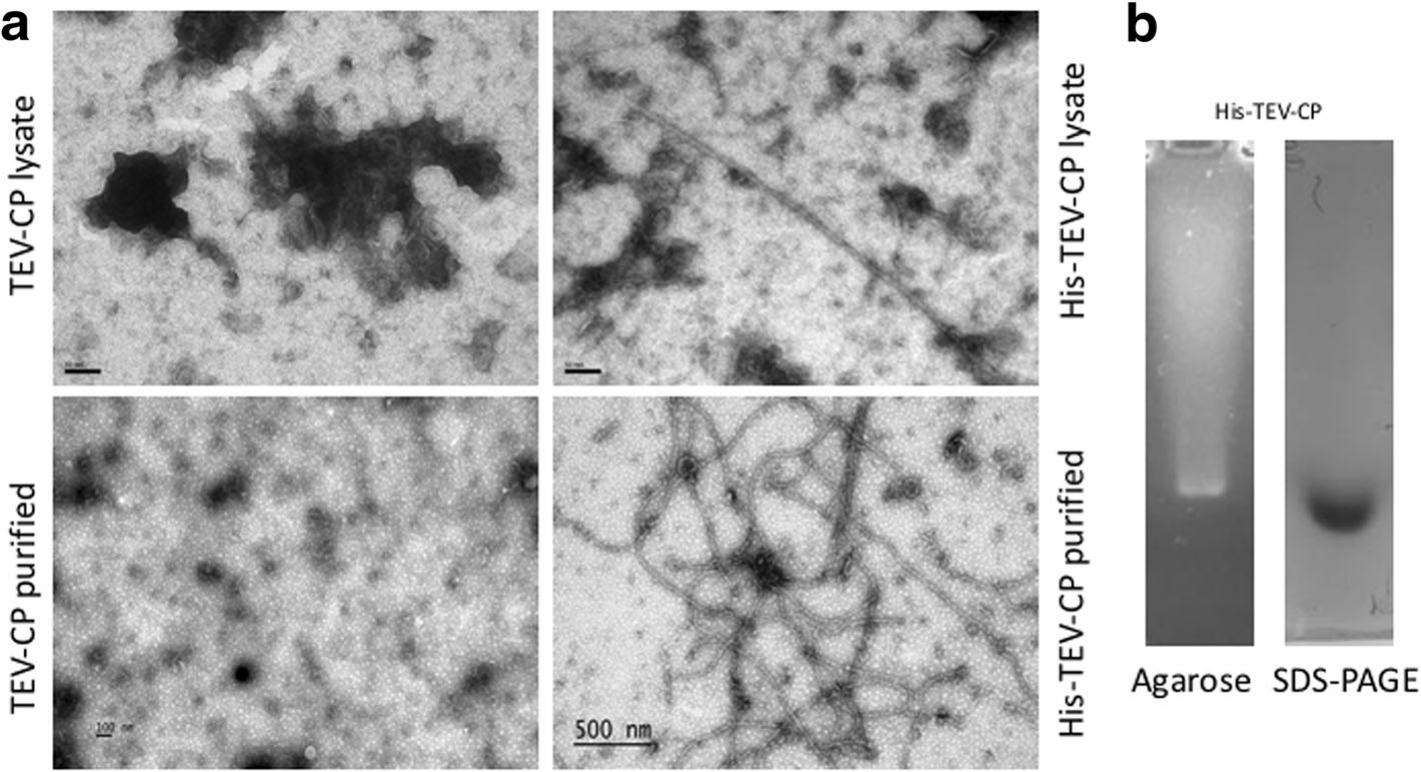
TEM analysis of VLP assembly of non-tagged and His-tagged TEV CP versions. **a** The samples were adsorbed on Formvar-coated copper grids and negatively stained with 2% uranyl acetate aqueous solution. The grids were examined using TEM JEM-100C (JEOL, Japan) at an accelerating voltage of 80 kV. TEV-CP lysate: TSP of TEV-CP preparation at 100,000x magnification; His-TEV-CP lysate: TSP of His-TEV-CP preparation at 100,000x magnification; TEV-CP purified: soluble fraction recovered from precipitation of TEV-CP extract using PEG 8000 3% and 500 mM NaCl at 50,000x magnification; His-TEV-CP purified: Ni^2+^ affinity purified His-TEV-CP (0.4 mg/ml) in MB, 500 mM NaCl at 50,000x magnification. **b** Visualization of nucleic acid content in purified His-TEV-CP VLPs sample analyzed in agarose gels. A sample of 25 μl of purified particle solution (0.2 mg/ml) was loaded in 0.8 agarose gel in Tris, acetic cid, EDTA (TAE) buffer. The particles were treated with Laemmli sample buffer (2x) for 10 min at 95 °C. Nucleic acids in gel were visualized using SYBR Safe DNA gel stain (Thermo Fisher Scientific, USA) included in the sample with Gel Doc EZ System (BioRad, USA) using a blue light tray. Proteins were identified with 0.1% Coomassie G-250 dye in 10% ethanol and 10% acetic acid and destained in the same solution without Coomassie dye

For assessing TEV CP VLPs formation, transmission electron microscopy (TEM) was performed (Fig. 2a). The micrographs revealed that His-tagged TEV CP version assembled into rod-shaped VLPs in the range of 20 nm to 2 μm, in contrast to non-tagged TEV CP version, where particles around 50 nm or shorter were observed. The mean length of His-tagged TEV CP VLPs was 553 nm. VLPs of less than 500 nm length represented 53% of all particles analyzed, while VLPs in the ranges of 500-1000 nm and more than 1000 nm represented 29.5 and 17.5%, respectively. Additionally, purified His-tagged TEV CP VLPs were analyzed for the presence of nucleic acids. For this, VLPs were denatured in protein loading buffer containing 2% sodium dodecyl sulfate, electrophoresed in agarose gel supplemented with SYBR-Safe (Thermo Fisher Scientific, USA) and nucleic acids were visualized with Gel Doc EZ System (BioRad, USA) using a blue light tray; same gel was subsequently stained with Coomassie brilliant blue solution for protein detection. Figure 2b shows the presence of nucleic acids that co-localize with TEV CP protein, which is typical of viruses and VLPs and is consistent with the findings of Kalnciema et al. [15], who found CP mRNA, 16S rRNA and 23S rRNA in *E. coli*-expressed potato virus M (PVM) VLP preparations. Nonetheless, these results must be taken carefully, because the possibility that RNA binds to the nickel-charged column used in this work to purify His-tagged TEV CP can not be ruled out [16].

**Fig. 3:**
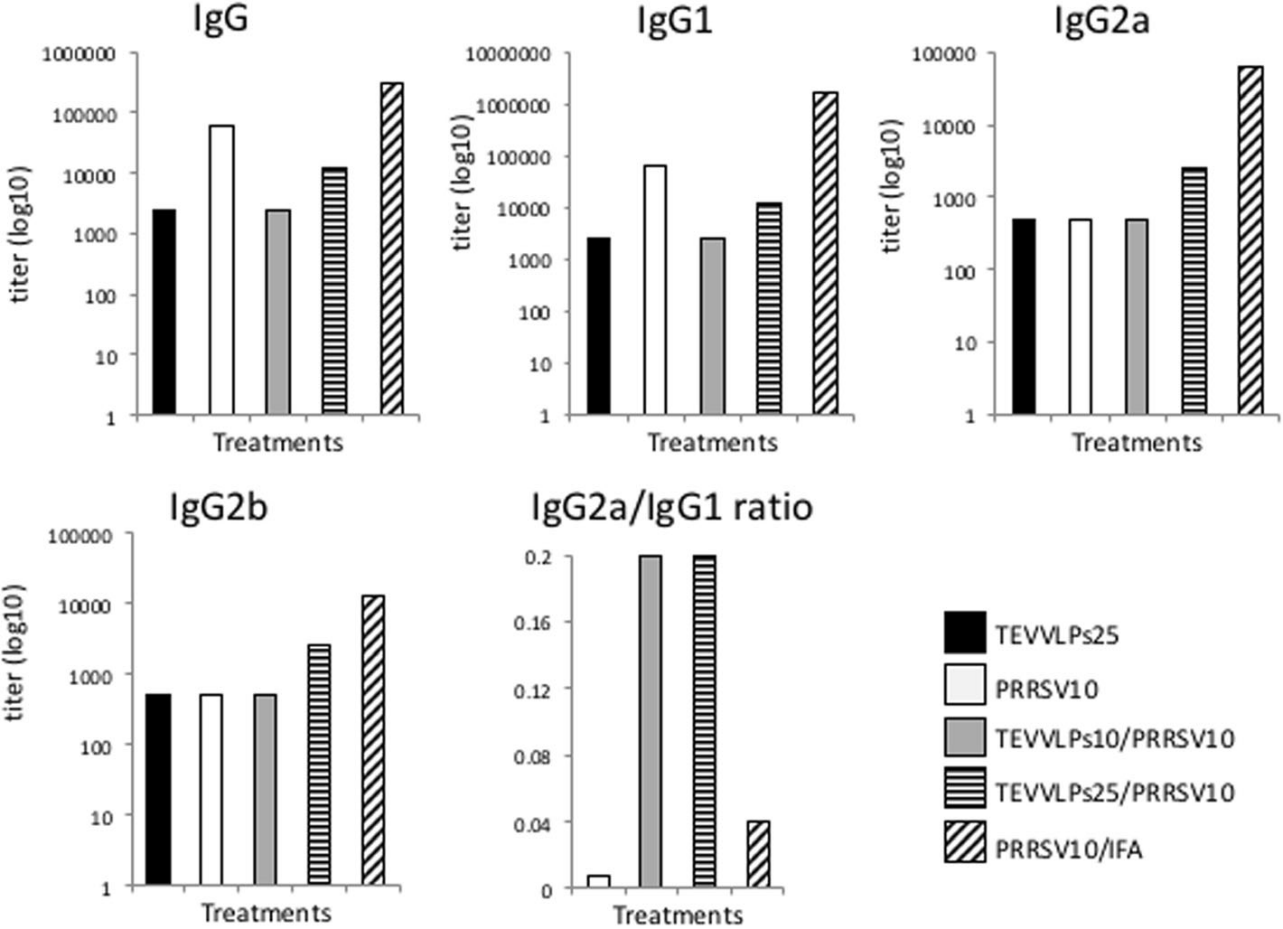
Antibody titers produced in sera of immunized mice recognizing PRRSV_chim_ protein. Groups were immunized with 25 μg of TEV VLPs (TEVVLPs25), 10 μg of PRRSV_chim_ protein (PRRSV10), 10 μg of TEV VLPs plus 10 μg of PRRSV_chim_ protein (TEVVLPs10/PRRSV10), 25 μg of TEV VLPs plus 10 μg of PRRSV_chim_ protein (TEVVLPs25/PRRSV10) or 10 μg of PRRSV_chim_ protein plus incomplete Freund’s adjuvant (PRRSV10/IFA). Immunization was carried out via subcutaneous with 100 μl of the different preparations on days 1 and 14. Blood samples were collected by tail vein bleeding before immunization and on day 28 after first immunization. Preimmune serum samples were checked for seronegativity to PRRSV_chim_ protein. Serum samples collected from the second bleeding were tested for IgG, IgG1, IgG2a and IgG2b antibody response to PRRSV_chim_ protein by ELISA. Individual samples belonging to the same group were pooled and diluted 1:20 followed by serial 1:5 dilution in blocking buffer. 96-well MaxiSorp Immuno Plates (Nunc, USA) were coated with 1 μg of PRRSV_chim_ protein per well diluted in carbonate buffer, blocked and washed. Diluted serum samples were added to the appropriate well. After incubation and washing steps, the appropriate secondary antibody was added, HRP-donkey anti mouse IgG (Bio-Techne, USA) diluted 1:1,000 or HRP-goat anti mouse IgG subclass 1, 2a or 2b (JIR Laboratories, USA) diluted 1:5,000. After incubation and washing steps, 3-3′, 5-5′ tetrametylbenzidine (TMB) solution (Sigma-Aldrich, USA) was added to each well. Color development was monitored before saturation and stopped using sulfuric acid. Absorbance was taken at 450 nm in xMark spectrophotometer microplate reader (BioRad, USA). Results are expressed as antibody endpont titers greater than threefold the background value of preimmune sera

In order to evaluate the adjuvant effect of TEV CP VLPs, a novel *E. coli*-expressed PRRSV chimeric protein, named PRRSV_chim_, was used as antigen. This protein comprises PRRSV GP3 and GP4 epitopes, GP5 and M ectodomains and thioredoxin as fusion partner. This chimeric protein was developed by our group for swine vaccination against this virus and all its details are included in a different manuscript that is under preparation. Twenty-five six-week-old female BALB/c mice (Harlan Laboratories, USA) were divided equally into 5 groups immunized with 25 μg of His-TEV-CP (TEV VLPs25), 10 μg of PRRSV_chim_ (PRRSV10), 10 μg of His-TEV-CP plus 10 μg of PRRSV_chim_ (TEV VLPs10/PRRSV10), 25 μg of His-TEV-CP plus10 μg of PRRSV_chim_ (TEV VLPs25/PRRSV10), or 10 μg of PRRSV_chim_ plus incomplete Freund’s adjuvant (PRRSV10/IFA). Immunization protocol as well as ELISA procedure for IgG, IgG1, IgG2a and IgG2b isotype determinations against PRRSV_chim_ were performed as described by Guerrero-Rodríguez and others [14]. As depicted in Fig. 3, PRRSV_chim_-specific IgG and IgG1 isotype determinations showed an increase in the antibody response elicited by PRRSV_chim_. However, TEV CP VLPs plus PRRSV_chim_ formulations induced an IgG and IgG1 response against the PRRSV_chim_ protein 5-25 times lower than PRRSV_chim_ treatment. Conversely, PRRSV_chim_ induced a low response of IgG2a and IgG2b isotypes, similar to the non-specific response to TEV CP VLPs. In addition, the IgG2a/IgG1 ratio against PRRSV_chim_ protein was 125 times higher in TEV CP VLPs plus PRRSV_chim_ formulations than PRRSV_chim_ itself. The antibody response against TEV CP VLPs was determined as well (Additional file 1: Figure S1), with IgG1, IgG2a and IgG2b titers of 12500, 2500 and 2500, respectively. This antibody profile differs from the one that resulted after administering native TEV particles through the intraperitoneal route [5], where the subclass that presented the highest increase was IgG2a. The different immunization routes used might explain these results. Others have reported that VLPs based on the HIV-1 Pr55gag precursor protein induce a predominant IgG1 subclass increase after subcutaneous injection [17].

Most of the research in VLP production and assembly has been focused in plant viruses with isodiametric symmetry, like Cowpea mosaic virus and Cowpea chlorotic mottle virus, the rigid rods of Tobacco mosaic virus (TMV) and the filamentous particles of Papaya mosaic virus (PapMV) [4]. The production of TEV CP VLPs has been previously accomplished in a previous work [13], where particles ranging from 90 to 750 nm in length were observed, but level of expression was not reported. Furthermore, the assembly of His-tagged versions of TEV CP into VLPs was not addressed, in contrast to the results shown here. There are two factors that can explain these disparate results: the plasmids used for expressing His-tagged TEV CP versions and expression levels. The previous work describes the use of a plasmid (pTrcHis B from Thermo Fisher Scientific, USA) that adds at least 35 amino acids, including the His-tag, to the N-terminus of the recombinant protein, compromising VLP formation, while the plasmid used in the present work (pET28a + from Merck-Millipore, Germany) just adds 8 amino acids to TEV CP, leaving VLP assembly unaffected. Regarding the non-tagged versions, it is possible that the expression levels of TEV CP in the previous report reached the minimum amount required for spontaneous assembly, which was not the case in the present work. We hypothesized that His-tagged TEV CP, expressed at moderately higher levels than its non-tagged counterpart, must have been assembled right after the elution step of Ni^2+^ affinity chromatography. This could explain why a few VLPs were observed by TEM when analyzing His-tagged TEV CP samples before purification (Fig. 2a), however, additional experiments must be carried out in order to confirm this hypothesis. To our knowledge, there is no work thus far describing the expression of a His-tagged potyviral CP that assembles into VLPs. TMV studies have demonstrated a pivotal role of His-tag on controlling assembly of CP monomers to different virus-like structures, like rings, discs, rods, and filamentous structures [18, 19]. However, despite the potential of affinity tags, like hexahistidine, to facilitate process development of proteins, these may have undesired consequences on protein stability in solution and immunogenicity [20].

The novel PRRSV_chim_ demonstrated to elicit potent IgG antibody response without the need of any adjuvant. This high immunogenicity is perhaps due to the ability of this chimeric protein to form oligomeric structures, as demonstrated by dynamic light scattering (Additional file 2: Figure S2), revealing the presence of a major peak with a mean diameter of 180 nm. However, the response is restricted to IgG1 subclass. As an unexpected result, this antibody response against PRRSV_chim_ did not increase when the TEV CP VLPs were administered along with, even when different ratios of VLPs were used (Fig. 3). In contrast, TEV CP VLPs induced a change in the IgG2/IgG1 ratio, counteracting the IgG1-polarized antibody response to PRRSV_chim_ protein alone (Fig. 3). In mice, it is well documented that IgG1 is related to Th2 immunity, while IgG2a reflects a Th1 bias. In addition, soluble proteins are generally restricted to the IgG1 isotype, while most antiviral response belongs to IgG2a [21]. PapMV CP nanoparticles administered with trivalent flu vaccine showed an increase in the production of IgG1 and IgG2a against influenza virus [22]. In addition, PRSV CP filamentous particles administered with green fluorescent protein (GFP) induce an increase in GFP-specific IgG1 and the strength of the response depends on the ratio of VLPs used [14]. Based on PRRSV_chim_ antibody response, it appears that TEV CP VLPs have a role in balancing both arms of the immune system, humoral and cellular response, against PRRSV_chim_. Some authors have mentioned that the success of a PRRSV vaccine relies on its ability to induce both neutralizing epitopes and cellular response [23]. To our knowledge there is no report in the use of TEV CP-based particles, some of them longer than 1 μm, for aiding the immune response against a soluble antigen. Nevertheless, an interesting work by Kalnciema et al. demonstrates the usefulness of PVY microparticles as antigen carriers when translationally fused to foreign sequences of up to 71 amino acids long [24]. Finally, it is becoming evident that the size of the adjuvant may have different effects on the type of the immune response induced; it appears that microparticles promote humoral response, whereas nanoparticles may favor the induction of cellular response [25]. In summary, our results demonstrate that a hexahistidine-tagged TEV CP is successfully purified by Ni^2+^ affinity chromatography and self-assembles into long VLPs. Also, these particles contribute in balancing the immune response elicited by a novel chimeric protein comprising sequences from a swine virus, which in turn is a potent immunogen *per se*. The immune response of swine immunized with these TEV CP VLPs/PRRSV_chim_ formulations needs to be investigated. These findings highlight the potential of TEV CP VLPs as particulate adjuvants with immune modulation properties without the need of antigen’s attachment.

## Additional files

Additional file 1: Figure S1. Antibody titers produced in sera of immunized mice recognizing His-TEV-CP protein. Immunization and ELISA procedures were conducted as described in Fig. 3 legend. Briefly explained, serum samples corresponding to bleeding 3 of mice group immunized with 25 μg of TEV VLPs (TEVVLPs25) were tested for total IgG and isotypes IgG1, IgG2a and IgG2b antibody response by ELISA in plates coated with with 1 μg of His-TEV-CP protein per well. Absorbance was taken at 450 nm in xMark spectrophotometer microplate reader (BioRad, USA). Results are expressed as antibody endpont titers greater than threefold the background value of preimmune sera. Excel was used for data processing. (TIFF 1142 kb)
Additional file 2: Figure S2. The size distribution of purified PRRSV_chim_ proteins was estimated using dynamic light scattering. a) Measurements were taken at 25 °C and at a nanoparticle concentration of 0.2 mg/ml in Tris-HCl 20 mM, pH 8.0, NaCl 500 mM. Recording and data analysis were taken in automated mode with the ZetaSizer Nano ZS90 and Software version 7.11 (Malvern, United Kingdom) using the 90° scattering optics. b) Analysis of purified PRRSV_chim_ by 12% SDS-PAGE/Coomassie staining. (TIFF 1142 kb)
