# Supplementary figures and images for: Self-assembly of hexahistidine-tagged tobacco etch virus capsid protein into microfilaments that induce IgG2-specific response against a soluble porcine reproductive and respiratory syndrome virus chimeric protein
Source: Virol J. 2016 Nov 29;13:196. doi: 10.1186/s12985-016-0651-y (PMC5126848; doi:10.1186/s12985-016-0651-y)

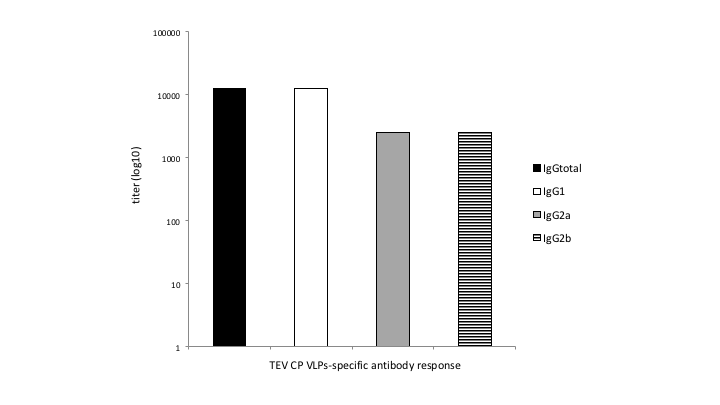

Supplement: Additional file 1: Figure S1. — Antibody titers produced in sera of immunized mice recognizing His-TEV-CP protein. Immunization and ELISA procedures were conducted as described in Fig. 3 legend. Briefly explained, serum samples corresponding to bleeding 3 of mice group immunized with 25 μg of TEV VLPs (TEVVLPs25) were tested for total IgG and isotypes IgG1, IgG2a and IgG2b antibody response by ELISA in plates coated with with 1 μg of His-TEV-CP protein per well. Absorbance was taken at 450 nm in xMark spectrophotometer microplate reader (BioRad, USA). Results are expressed as antibody endpont titers greater than threefold the background value of preimmune sera. Excel was used for data processing. (TIFF 1142 kb) [file 12985_2016_651_MOESM1_ESM.tiff]

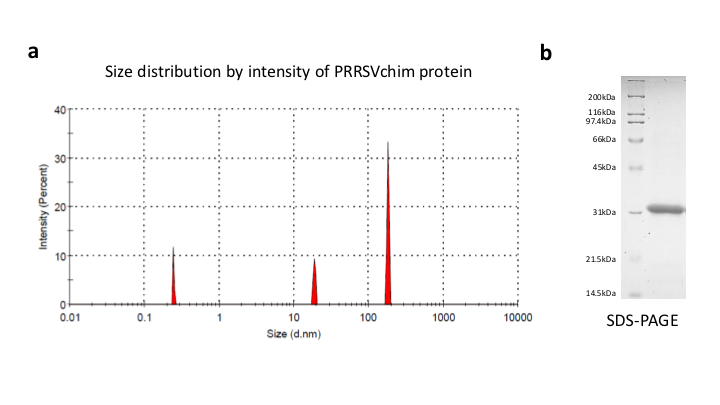

Supplement: Additional file 2: Figure S2. — The size distribution of purified PRRSVchim proteins was estimated using dynamic light scattering. a) Measurements were taken at 25 °C and at a nanoparticle concentration of 0.2 mg/ml in Tris-HCl 20 mM, pH 8.0, NaCl 500 mM. Recording and data analysis were taken in automated mode with the ZetaSizer Nano ZS90 and Software version 7.11 (Malvern, United Kingdom) using the 90° scattering optics. b) Analysis of purified PRRSVchim by 12% SDS-PAGE/Coomassie staining. (TIFF 1142 kb) [file 12985_2016_651_MOESM2_ESM.tiff]
